# For more than money: willingness of health professionals to stay in remote Senegal

**DOI:** 10.1186/s12960-019-0363-7

**Published:** 2019-04-25

**Authors:** Ayako Honda, Nicolas Krucien, Mandy Ryan, Ibrahima Ska Ndella Diouf, Malick Salla, Mari Nagai, Noriko Fujita

**Affiliations:** 1 Department of Economics, Sophia University, 7-1 Kioi-cho, Chiyoda-ku, Tokyo, 102-8554 Japan; 2 Health Economics Research Unit, University of Aberdeen, Scotland, UK; 3 Ministère de la Santé et de l’Action Sociale du Sénégal, Dakar, Senegal; 4 National Centre for Global Health and Medicine, Tokyo, Japan

**Keywords:** Rural job retention, Human resources for health, Motivation, Incentives, Low- and middle-income countries, Senegal, Discrete choice experiment

## Abstract

**Background:**

Poor distribution of already inadequate numbers of health professionals seriously constrains equitable access to health services in low- and middle-income countries. The Senegalese Government is currently developing policy to encourage health professionals to remain in areas defined as ‘difficult’. Understanding health professional’s preferences is crucial for this policy development.

**Methods:**

Working with the Senegalese Government, a choice experiment (CE) was developed to elicit the job preferences of physicians and non-physicians. Attributes were defined using a novel mixed-methods approach, combining interviews and best-worst scaling (Case 1). Six attributes were categorised as ‘individual (extrinsic) incentive’ attributes (‘type of contract’, ‘provision of training opportunities’, ‘provision of an allowance’ and ‘provision of accommodation’) or ‘functioning health system’ attributes (‘availability of basic equipment in health facilities’ and ‘provision of supportive supervision by health administrators’). Using face-to-face interviews, the CE was administered to 55 physicians (3909 observations) and 246 non-physicians (17 961 observations) randomly selected from those working in eight ‘difficult’ regions in Senegal. Conditional logit was used to analyse responses. This is the first CE to both explore the impact of contract type on rural retention and to estimate value of attributes in terms of willingness to stay (WTS) in current rural post.

**Results:**

For both physicians and non-physicians, a permanent contract is the most important determinant of rural job retention, followed by availability of equipment and provision of training opportunities. Retention probabilities suggest that policy reform affecting only a single attribute is unlikely to encourage health professionals to remain in ‘difficult’ regions. The relative importance of an allowance is low; however, the level of such financial incentives requires further investigation.

**Conclusion:**

Contract type is a key factor impacting on retention. This has led the Senegalese Health Ministry to introduce a new rural assignment policy that recruits permanent staff from the pool of annually contracted healthcare professionals on the condition that they take up rural posts. While this is a useful policy development, further efforts to retain rural health workers, considering both personal incentives and the functioning of health systems, are necessary to ensure health worker numbers are adequate to meet the needs of rural communities.

## Background

The health workforce plays a key role in healthcare service delivery. Equitable distribution of a quality health workforce contributes to ensuring the availability of healthcare services, irrespective of location, and to progressing towards the Universal Health Coverage (UHC) goal by facilitating access to quality healthcare services to all [1, 2].

In most countries, the geographical distribution of health workers is skewed towards urban and wealthier areas [3]. While approximately one half of the global population lives in rural areas [4], the rural population are served by only one quarter of the world’s doctors and by less than one third of the world’s nurses [5]. Inequitable geographical distribution of the health workforce has more severe implications for low- and middle-income countries (LMICs), which suffer from critical shortages of doctors, nurses and midwives [2]. The 36 sub-Saharan African countries bear approximately 24% of the global burden of disease and have only 3% of the global health workforce [3].

The inequitable distribution of already inadequate numbers of qualified health workers is a critical barrier to providing health services in LMICs and is often a serious constraint to ensuring fair access to essential health services and achieving health system goals. Links between the number of health workers in a country and both service delivery and health outcomes have been clearly demonstrated [5, 6]. Consequently, while the issue of geographical health inequity is multidimensional, requiring consideration of both the number of health workers and the quality of services [6], the absolute number of health workers in rural and remote areas in LMICs is low, and concerted efforts are required to address health worker retention in those areas to create a better geographical balance in the distribution of skilled health workers [1].

Senegal is a lower middle-income country, located in sub-Saharan Africa, with a population of 15.9 million [4]. Rural residents accounted for 55.6% of the population in 2017, slightly decreasing from 58.5% in 2007 [4]. In Senegal, the physician to population ratio was 0.1 per 1000 people and the ratio for nurses and midwives was 0.3 per 1000 people in 2016 [7]. The figures are lower than sub-Saharan African averages (0.3 physicians per 1000 people; 1.1 nurses and midwives per 1000 people in 2016) and countries with a similar economic status (1.6 physicians per 1000 people; 2.3 nurses and midwives per 1000 people in 2016) [8]. The shortage of health workers is even more severe in rural Senegal [9]. In 2012, 66% of all physicians in Senegal (667 of 1011 physicians) were located in the Dakar region, which houses the nation’s capital [5], while 76% of the population live outside Dakar [4]. In addition to the geographical inequitable distribution of inadequate numbers of health professionals, Senegal’s health system also suffers from widespread health professional absenteeism and poor-quality healthcare services [10].

Over the past decade, the Senegalese Health Ministry has made efforts to address the inequitable distribution of qualified health professionals, including the introduction of measures to improve posting and recruitment processes for health workers in rural and remote areas [9]. Currently, the Senegalese Government is developing policy that aims to encourage health professionals to remain in rural posts, particularly in areas that the Government defines as ‘difficult’ regions. The Human Resources Department of the Senegalese Health Ministry has a working definition of ‘difficult’ regions which includes geographical areas that constrain professional, personal and family growth and are characterised by a set of geographical, security, infrastructure and social service criteria [11]. While there is political momentum to improve conditions for those in ‘difficult’ regions, the Government is restricted by poor resource availability and hopes to identify priority areas for reform. This study determines how different aspects of working conditions encourage health workers to stay in rural areas.

## Methods

The study employed the choice experiment (CE) methodology. This approach is increasingly used to elicit health preferences in a range of areas [12], including health worker preferences [13]. CEs ask individuals to state preferences for hypothetical alternatives, each described by several attributes. They are a favoured technique in preference research because they allow estimation not just of what is important, but how important it is. By asking individuals to make trade-offs between attributes, and analysing responses in a random utility framework, researchers can estimate marginal rates of substitution between attributes (MRS; how much of one attribute is needed to compensate for the reduction in another attribute) and the probability accepting a given job.

This study extends the current literature applying CE to job choices in LMICs in two ways. Firstly, a novel mixed-methods approach was used to develop attributes and levels, combining interviews with a best-worst scaling (BWS) (Case 1) experiment. This is the first study to use the BWS (Case 1) method to reduce the number of attributes to a manageable level. Secondly, a ‘period of assignment’ attribute was included to estimate trade-offs, allowing the influence of other attributes on intended time in a post to be determined. To the authors’ knowledge, this is the first study to include such an attribute to estimate trade-offs.

### Defining attributes and levels

A novel two-stage approach was used to develop attributes and levels. Interviews were first undertaken with 176 healthcare professionals working in remote, rural and urban areas (31 physicians, 94 nurses, 51 midwives) to explore factors influencing the retention of healthcare professionals in rural posts [14]. In-depth interviews with eight health administrators in the Senegalese Health Ministry were also undertaken. Thematic analysis identified an initial list of factors that motivated or demotivated healthcare workers in rural areas. The factors were categorised as pre-service and in-service education, regulatory systems, financial and non-financial incentive schemes and professional and personal support. Subsequent analysis identified 14 factors that influenced the retention of healthcare professionals in rural posts.

**Fig. 1:**
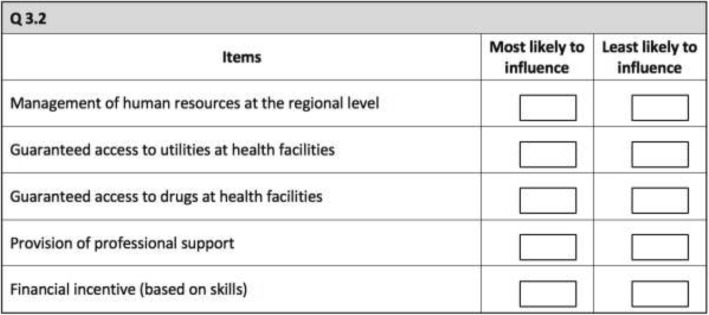
Example of best-worst task

A BWS (Case 1) approach [15, 16] was used to reduce the 14 attributes to a manageable number for use within a CE (5–7 attributes) [12]. Respondents to the BWS study were 266 health professionals, comprising 170 nurses, 68 midwives and 28 clinicians working in the ‘difficult’ regions of Senegal. Locally trained interviewers administered the best-worst tasks using face-to-face interviews. Respondents were given 14 best-worst tasks (Fig. 1). In each task, respondents were asked to select the attributes that were most and least likely to influence their decisions to stay in rural posts.

The BWS data were analysed using both count and choice analysis, and the results were compared to confirm validity [15, 16]. The results from the count analysis are shown in Table 1. The choice analysis results were consistent with the count analysis and are available from the authors upon request. Items ranked highly in the BWS were proposed for inclusion in the CE, with careful consideration given to whether the items were relevant to both policy and the working conditions of physicians and non-physicians in Senegal. Specifically, the five most valued items in the BWS were considered for inclusion as attributes in the CE. A series of discussions with team members who were experienced in data collection helped to clarify and refine the items from the BWS for use in the CE. For example, ‘support for career development’ used in the BWS was discussed in the context in Senegal to develop an implementable policy action, and re-defined as ‘provision of training opportunities’ (the detailed definition of a training opportunity is provided in Table 2). Also, as discussed later in this section, the inclusion of the period of assignment attributes was discussed together with the BWS results to establish the final set of attributes. Discussion with the Health Ministry assisted in the finalisation of the attributes and levels (Table 2) for use in the CE.

**Table 1:** Count analysis of best-worst data at a sample level

Item	Best	Worst	Ratio score	Rescaled ratio score
Improved professional mobility	124	365	− 0.540	0.189
Management of human resources at the regional level	97	410	− 0.721	0.077
Promote participation in social events	251	297	− 0.084	0.470
Development of inter-professional exchange	69	375	− 0.846	0.000
Help to get scholarship	195	365	− 0.313	0.328
Help to get public contract	596	126	0.777	1.000
Provision of professional support	272	227	0.090	0.577
Financial incentive (based on distance)	644	177	0.646	0.919
Financial incentive (based on skills)	273	403	− 0.195	0.401
Provision of accommodation	316	220	0.181	0.633
Guaranteed access to medical equipment	345	169	0.357	0.741
Guaranteed access to drugs	140	154	− 0.048	0.492
Guaranteed access to utilities	146	223	− 0.212	0.391
Support of career development	258	214	0.094	0.579
Individuals	266			
Best	3 726/3 738			
Worst	3 725/3 738

**Table 2:** Attributes and levels

Attribute	Category	Definition	Regression labels	Levels
Period of assignment	Individual benefits	The total number of years of assignment to a rural/remote job	PERIOD	1. 2 years 2. 4 years 3. 6 years 4. 8 years
Allowance	Individual benefits	Provision of skills/qualification-based allowance or rural/remote job allowance	ALLOWANCE	1. No allowance 2. Rural job allowance provided 3. Skill-based allowance provided (for physicians)
Equipment	Functioning of health system	Availability of equipment at the health facility that allows the provision of a basic package of health care services	EQUIP	1. Inadequate: Medical equipment at the facility does not allow the provision of a basic package of health services 2. Adequate: Medical equipment at the facility allows the provision of a basic package of health services
Accommodation	Individual benefits	While working in a rural area, the employer provides free accommodation that is appropriate for marital/family status	ACCOMMOD	1. No provision of accommodation 2. Accommodation provided
Types of contract	Individual benefits	Either permanently contracted government workers, temporary contract with MoH; temporary contract with health facilities; or temporary contract with local authorities	CONTRACT	1. Permanent: permanently contracted government workers 2. Temporary (MoH): Temporary appointment by MoH 3. Temporary (Health facility): Temporary appointment by health facilities 4. Temporary (Local): Temporary appointment by local authorities
Training opportunities	Individual benefits	Provision of further training offered outside the work place (excluding further education for degree purposes)	TRAINING	1. No provision of training opportunities 2. Training opportunities provided
Support (for non-physicians)	Functioning of health system	Either no support; supportive supervision by health administrators; or clinical advice and support from peer health professionals	SUPPORT	1. No support 2. Managerial support: Supportive supervision by health administrators 3. Clinical support: Clinical advice and support from peer health professionals

The CE attributes were classified as factors relating to (1) *individual (extrinsic) incentive benefits* (type of contract, provision of training opportunities, provision of an allowance, and provision of accommodation) and (2) *functioning of health systems* (availability of basic equipment in health facilities and provision of supportive supervision by health administrators). As the working conditions for physicians and non-physicians can be diverse, including in the types of allowance provided (e.g. physicians receive a skills-based allowance, non-physicians receive a rural allowance), different CEs were given to each group of healthcare workers.

Most CEs eliciting job preferences use a salary attribute to determine how much health workers need compensating to accept a reduction in working conditions, i.e. working in a rural rather than urban location [17]. However, discussions with local interviewers and staff in the Senegalese Health Ministry revealed that salary-related questions were culturally sensitive and could make respondents feel uncomfortable and reluctant to respond to the survey. Furthermore, the Health Ministry did not plan to increase the salaries of health professionals, except through the provision of a rural allowance. Consequently, we used an assignment period attribute, number of years of assignment to a rural post, to determine the ‘willingness to stay’ (WTS, i.e. how long health workers would stay in difficult areas if certain working conditions were improved). To the best of our knowledge this approach has not been previously employed in LMICs.

### Defining choice tasks

**Fig. 2:**
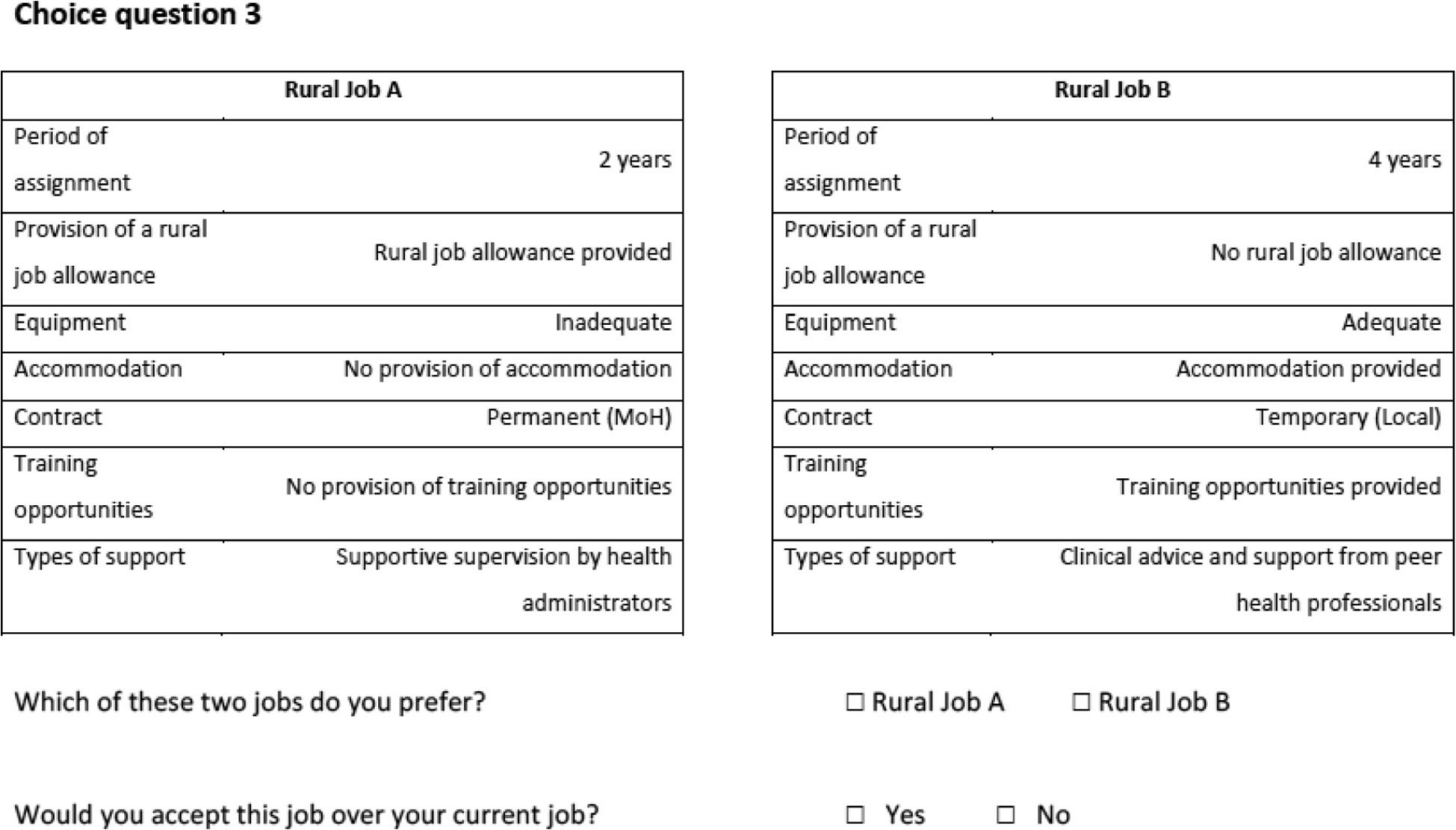
Example of choice task

A D-efficient design was used to identify the choice tasks to present to respondents. This approach minimises the standard errors (SEs) of parameters [18], thus ensuring more precise parameter estimates. Assuming null interaction effects between attributes (i.e. the preferences for one attribute do not depend on the level of another attribute) and using non-informative priors (no a priori information on preference parameters), the approach generated 15 choice tasks for physicians and 16 choice tasks for non-physicians. Each choice task presented two rural job options and asked respondents to choose their preferred option. A subsequent question asked if they would prefer to remain in their current position rather than take up the chosen option (opt-out response). Figure 2 presents an example choice task. The questionnaire also collected information on socio-demographic characteristics of respondents. A copy of the questionnaire is available from the authors upon request.

### Sample, setting and data collection

The study elicited the job preferences of physician and non-physician health workers (specialised nurses, nurses and midwives). Data collection took place in the eight ‘difficult’ regions of Senegal (of a total of 14 regions). Using the Health Ministry’s human resources database, participants were randomly selected from a list of physician and non-physician health professionals working in clinics and hospitals in the regions of interest. If a pre-determined health professional was unavailable, or had been transferred, a health worker of the same professional type and at the same health facility was substituted in their place. Louviere et al’s [19] sample size calculator determined that a minimum of 42 physician and 44 non-physician participants was necessary for the study (choice probability = 40%, confidence level = 95%, accuracy level = 90%, attrition rate = 10%, number of tasks = 15 or 16).

Face-to-face interviews were used to collect data [20]. A locally trained team of 10 interviewers and two field coordinators collected the data. Prior to the commencement of data collection, the questionnaire was pilot tested on 16 respondents in two health centres. After the pilot testing, the definitions of some attributes were changed and the levels of some attributes revised.

### Data analysis

Data from the CE allowed ranking of the three jobs (jobs A, B, and current), as well as estimation of the probability of a particular job being best (ranked first) or worst (ranked last). We applied partial rank ordering when a respondent answered A (or B) in both questions, with only the best choice data used. This approach maximised the information obtained from the CE.

The data was analysed within the random utility maximisation (RUM) framework [21, 22], which assumes that in each choice (*t* = 1,…,*T*), health professionals (*n* = 1,…,*N*) derive utility (*U*_*ntj*_) for a job (*j* = 1,…,*J*) and choose the position yielding the highest utility, subject to errors (*ε*_*ntj*_*).* Assuming errors are independently and identically distributed as type 1 extreme values (IID EV1), the choice model takes the form of a multinomial logistic (MNL) regression.

The regression equation for physicians is:

$$ {U}_{ntj}^{\mathrm{PHYS}}={\beta}_0{\mathrm{CURRENT}}_{ntj}+{\beta}_1\mathrm{ALLOWANCE}\_{1}_{ntj}+{\beta}_2\mathrm{ALLOWANCE}\_{2}_{ntj}+{\beta}_3{\mathrm{EQUIP}}_{ntj}+{\beta}_4{\mathrm{ACCOMOD}}_{ntj}+{\beta}_5\mathrm{CONTRACT}\_{1}_{ntj}+{\beta}_6\mathrm{CONTRACT}\_{2}_{ntj}+{\beta}_7\mathrm{CONTRACT}\_{3}_{ntj}+{\beta}_8{\mathrm{TRAINING}}_{ntj}+{\beta}_9{\mathrm{PERIOD}}_{ntj}+{\varepsilon}_{ntj} $$

And the regression equation for non-physicians is:

$$ {U}_{ntj}^{\mathrm{NOPHYS}}={\beta}_0{\mathrm{CURRENT}}_{ntj}+{\beta}_1{\mathrm{ALLOWANCE}}_{ntj}+{\beta}_2{\mathrm{EQUIP}}_{ntj}+{\beta}_3{\mathrm{ACCOMOD}}_{ntj}+{\beta}_4\mathrm{CONTRACT}\_{1}_{ntj}+{\beta}_5\mathrm{CONTRACT}\_{2}_{ntj}+{\beta}_6\mathrm{CONTRACT}\_{3}_{ntj}+{\beta}_7{\mathrm{TRAINING}}_{ntj}+{\beta}_8{\mathrm{PERIOD}}_{ntj}+{\beta}_9\mathrm{SUPPORT}\_{1}_{ntj}+{\beta}_{10}\mathrm{SUPPORT}\_{2}_{ntj}+{\varepsilon}_{ntj} $$

where all regression labels are defined in Table 2.

Willingness to stay (WTS) for marginal improvements in attributes, calculated as the ratio of the coefficient of interest to the negative of the coefficient on the assignment period attribute, was estimated. Associated confidence intervals were computed for all attributes using the delta method [23]. Overall WTS for a defined job was calculated as the sum of the WTS values for the job’s various features.

Results from the MNL regression model were used to predict the probability of health workers remaining (*P*_*ntj*_) in a pre-defined (baseline) rural job (*j*).

$$ {P}_{ntj}=\frac{\exp \left({U}_{ntj}-{\varepsilon}_{ntj}\right)}{\sum \limits_j\exp \left({U}_{ntj}-{\varepsilon}_{ntj}\right)} $$

The baseline scenario reflected current working conditions in the ‘difficult’ regions of Senegal: 4-year assignment period, no allowance, inadequate equipment at the health facility, no accommodation, temporary contract with the Health Ministry, no training, and no supportive supervision (non-physicians only). The retention rate for the baseline scenario was compared with retention rates for job contracts offering improvements in attributes.

## Results

### Respondent characteristics

The study included 55 physicians and 246 non-physicians. The physician group comprised 37 general practitioners (GPs) and 18 specialists, and the non-physician group comprised 153 nurses, 83 midwives and 11 specialised nurses. Of the physician respondents, 96.4% were male, while 60.2% of the non-physicians were female. The skewed gender distribution in physician respondents reflects actual patterns in the gender distribution of physicians working in the ‘difficult’ regions [7]. Health ‘posts’ are the smallest type of health facility operating in Senegal and are run by non-physicians. Consequently, health ‘posts’ did not have physician respondents. The amount of time spent working in rural/remote areas averaged 5.3 years for physician respondents (minimum less than 1 year; maximum 17 years; SD 4.3) and 7.4 years for non-physicians (minimum less than 1 year; maximum 38 years; SD 8.0). A range of contract types was used: 61.8% of physicians were permanently contracted government employees, 7.3% were annually contracted by the Health Ministry and 30.9% hired either by health facilities or local authorities. For the non-physicians, 60.7% were permanent government employees, 17.4% were annually contracted by the Health Ministry, and 21.9% were locally hired.

### Preferences for job attributes

All attributes, except professional support for non-physicians, were statistically significant, indicating that they impact on the probability of health professionals staying in a rural job (Table 3). The constant term for non-physicians was statistically significant with a negative coefficient, indicating a general preference not to remain in ‘difficult’ regions.

**Table 3:** Conditional Logit model

	Physician			Non-physician		
	Coefficient	SE^a^	*P* value	Coefficient	SE^a^	*P* value
1. Model parameters						
Current job condition	− 0.093	0.124	0.454	− 0.442	0.066	< 0.001
Period	− 0.195	0.026	< 0.001	− 0.172	0.016	< 0.001
Allowance	0.382	0.125	0.002	0.267	0.060	< 0.001
Equipment	0.732	0.166	< 0.001	0.577	0.066	< 0.001
Accommodation	0.274	0.149	0.065	0.180	0.061	< 0.001
Temporary with MoH	− 1.071	0.182	< 0.001	− 0.754	0.086	0.003
Contract with health facility	− 1.849	0.259	< 0.001	− 1.520	0.097	< 0.001
Contract with local authorities	− 1.873	0.202	< 0.001	− 1.459	0.113	< 0.001
Training	0.955	0.137	< 0.001	0.346	0.064	< 0.001
Managerial support	–	–	–	0.306	0.079	< 0.001
Professional support	–	–	–	0.043	0.071	0.542
2. Model statistics						
Respondents	55			246		
Observations	3 909			17 961		
Log likelihood	− 905.98			− 5 053.37		
Bayesian info criterion	1 886. 40			10 214. 49		

^a^Robust standard errors adjusted for clustering on individual participants

For physicians, a change in contract from temporary to permanent has the greatest impact on the probability of staying in a rural post, followed by the provision of training opportunities and the availability of equipment at a health facility. Similarly, for non-physicians, the provision of a permanent contract was the most valued attribute, followed by the availability of equipment and the provision of further training opportunities.

Both physicians and non-physicians ranked attributes relating to the functioning of health systems (availability of equipment at a health facility and the provision of supportive supervision) higher than some of the individual benefit attributes (provision of an allowance and accommodation).

### Willingness to stay (WTS) in ‘difficult’ regions

**Fig. 3:**
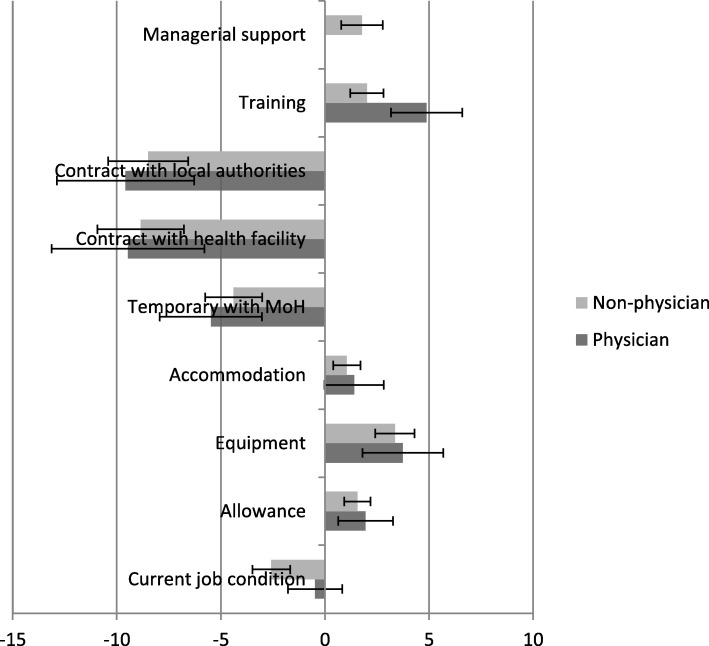
Willingness to stay (WTS) estimates

WTS for job improvements are shown in Fig. 3. WTS with improvements in the three most valued attributes (permanent contract, availability of equipment in health facilities and provision of further training opportunities) is 10.7 years for physicians and 5.4 years for non-physicians. However, if a permanent contract is not provided, and improvements only made to the availability of equipment and provision of training opportunities, the overall WTS is − 0.3 years for physicians and − 3.4 years for non-physicians. These negative figures suggest that, given the opportunity, both physicians and non-physicians would choose not to complete their assignments to the post.

### Probability of staying in ‘difficult’ regions

**Fig. 4:**
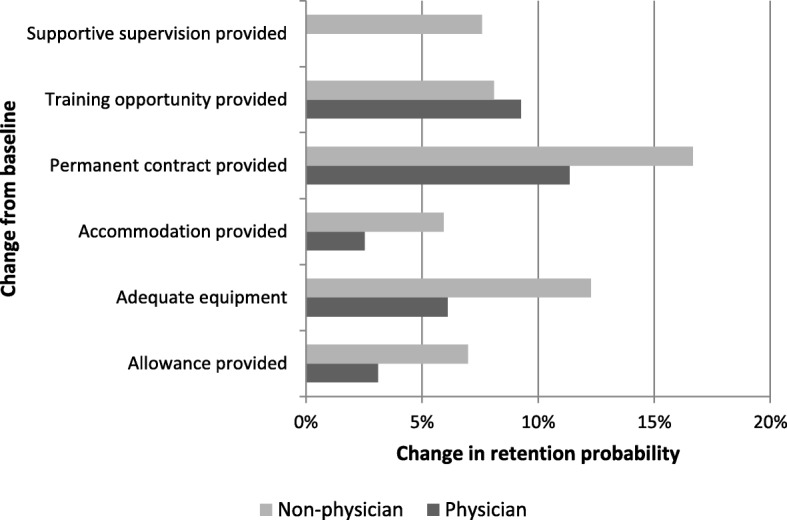
Effects of different policy options on retention probabilities

Figure 4 presents retention probabilities for different policy options. The baseline (current) job contract has a 1.5% probability of retaining physicians in the position for the 4-year assignment period and 4.2% probability of retaining non-physicians in current positions. Provision of a permanent contract increases the probability of retaining a physician to 11.4% and non-physicians to 16.7%. Availability of adequate equipment increases the retention rate to 6.1% for physicians and 12.3% for non-physicians. Further training opportunities increase the retention rate to 9.3% for physicians and 8.1% for non-physicians.

As with the WTS results, the retention probabilities suggest that policy reform affecting only a single attribute is unlikely to ensure health workers remain in ‘difficult’ regions, and retention policies should consider a combination of reforms. For instance, the retention rate for rural posts offering the three most preferred job conditions (permanent contract, availability of adequate equipment and further training opportunities) increases to 79.0% for physicians, an improvement of 77.5% points above the baseline, and 55.9% for non-physicians, an improvement of 51.7% points above the baseline.

Combinations of individual benefit incentives and aspects of health system functioning were also considered. The retention rates for rural posts offering the three most preferred individual benefit incentives (i.e. permanent contract, further training opportunities and provision of an allowance) are estimated to be 65.1% for physicians and 40.5% for non-physicians. These retention rates increase to 89.0% for physicians and 79.9% for non-physicians if factors relating to the functioning of health systems are also improved (i.e. availability of basic equipment at health facilities for physicians, and availability of basic equipment and provision of supportive supervision for non-physicians). This finding supports the importance of improving health system functioning to improve that likelihood of health professionals remaining in rural posts.

## Discussion

This study contributes to the literature on the retention of health workers in the rural areas of LMICs in a number of ways. The study contains two methodological originalities. Firstly, the study employed a BWS (Case 1) experiment to short-list factors identified in qualitative interview data and used a ranking score to determine policy options for inclusion in the CE. While most of the recent CE studies in LMICs have established attributes and assigned attribute levels using a qualitative approach, such as group discussions and in-depth interviews, this is the first CE in LMICs, and indeed CE in any context, which has applied BWS to finalise attributes after qualitative work was undertaken. BWS (Case 1) was a useful approach to reducing the number of attributes in a CE to a manageable level. Secondly, to ensure the cultural acceptance and policy relevance of the attributes, WTS, instead of willingness to pay, was estimated using the period of assignment to determine trade-offs. This allowed estimation of how long respondents would be willing to stay in a rural post if there were improvements in other aspects of the contract attributes.

At the applied level, while CEs have been extensively used to investigate human resource issues in sub-Saharan Africa [24–31], the number of CEs in Western Africa is limited. Indeed, our study is only the third in Western Africa [32, 33], and the first in Senegal. Our results highlight the challenges of retaining the health workforce in rural areas of Senegal. The statistically significant negative constant term for non-physicians suggests that health workers are unlikely to want to remain in current roles in ‘difficult’ regions for the term of their posts. Policy makers must promptly respond to rural job retention issues, being mindful that policy reforms addressing single attribute are unlikely to improve retention rates. Retention policy should include a combination of reforms, including both individual incentives and factors relating to how health systems function.

To the authors’ knowledge, this is the first CE study in LMICs that has included type of contract as an attribute, though two studies have included attributes on the number of years of service before obtaining a permanent post or promotion to permanent staff member [34, 35]. For both physicians and non-physicians, provision of a permanent contract was the factor which most affected the likelihood of job retention. In Senegal, while permanently contracted government workers, including public sector medical doctors and nurses, are employed and paid by the Ministry of Public Services, other health professionals are contracted annually by the Health Ministry [36]. Annually contracted health professionals do not receive government employee social benefit packages, such as pensions; however, the base salary for annually contracted health professionals is slightly higher than that of permanent health professionals. Renewal of annual contracts is unpredictable, depending on the availability of Health Ministry budget [36]. Given the key differences in the job conditions between the two types of contracts are the length of job security and the provision of social security entitlements, our study results suggest that respondents’ value stability in employment and/or the entitlements associated with permanent employment.

In 2006, a program called “Plan Cobra” was introduced in Senegal, enabling the Health Ministry to hire health professionals using annual contracts. The plan aimed to address human resource shortages, particularly in rural areas, in a timelier manner than the lengthy process of hiring government workers through the Ministry of Public Services. While Plan Cobra used annual contracts to help distribute human resources to rural posts when it started in 2006 [9], over time, the nature of the contracts with the Health Ministry changed and, currently, annual contracts are used to employ health professionals regardless of geographical location. While in 2016, 48.9% of public sector health professionals, including doctors, nurses and midwives, were permanent government employees [36], the proportion in our sample (healthcare professionals working in difficult regions) was around 60%. Our results suggest that a short-term contract policy will not be effective in rural retention of healthcare professionals in the context of Senegal. Indeed, the Health Ministry has used the results from this study to introduce a rural assignment policy to recruit permanent staff from the pool of annually contracted healthcare professionals on the condition that they are assigned to rural posts. The results of this new policy intervention in Senegal require on-going monitoring. An ‘emergency-hire’ project for the recruitment of rural staff in Kenya saw most of those hired through the scheme leave rural areas after they were absorbed into the Government of Kenya’s public service [37]. Such experiences in other contexts suggest that it is important to further examine the aspects of permanent contracts that facilitate the retention of healthcare professionals in rural areas.

Current evidence on effectiveness of monetary incentives (either in salary increases or bonus payments) on rural retention is mixed—while monetary incentives can enhance the motivation and retention of health professionals in rural and remote areas, provision of non-monetary incentives can be equally important [13, 38]. The results revealed that monetary incentives (rural or skills-based allowances) had a relatively small impact on retention in the study context. However, the study did not specify the level of the rural allowance to be provided. While a small rural allowance is likely to have little impact, a larger allowance may have a greater impact. Thus, further investigation is warranted. In addition, the qualitative study undertaken prior to the choice experiment indicated the importance of fair, transparent administration of salary and/or allowance payments [14], which also suggests that the study results on payment of allowances must be carefully interpreted and further investigation of various aspects associated with payment is required.

Our results show the importance of improving the functioning of health systems, which includes ensuring the availability of basic equipment at health facilities and the provision of supportive supervision. A number of studies have found that healthcare professionals strongly value the availability of equipment and infrastructure [39–41]. Given that less than 50% of the clinics in rural Senegal have access to basic equipment, and less than 30% of rural health facilities have access to electricity, water and sanitation [10], our results suggest that there will be difficulty in retaining health workers even if individual incentives are provided. Retention may be improved using innovative approaches at the community level if the government cannot find immediate solutions due to limited resource capacity [42].

Our results suggest a small proportion of staff want to see out their contracts under the current arrangements. This contradicts the actual longer periods that respondents have already served (averaging 5.3 and 7.4 years for physicians and non-physicians, respectively). Given the Senegalese context, where fiscal constraints can prevent the appointment of public sector health workers and where many health professionals are unemployed [36], this contradiction suggests employed healthcare professionals do not abandon their current posts, perhaps for fear of joining the ranks of the unemployed. Alternatively, those who have served long periods in difficult areas may be more able to cope, or have learned to cope, and so are more likely to stay even if their grievances are similar to those who have left the difficult areas. This requires further investigation.

Our CE was administered to those currently working in ‘difficult’ regions. It did not examine the preferences of those who had left rural posts or those studying to be health professionals. These groups may have different job preferences for work in rural posts than those interviewed in our study. Although this may limit the generalizability of the study results to all healthcare professionals in Senegal, a qualitative study undertaken prior to the CE, which included interviews with those currently working in Dakar who had previously worked in ‘difficult’ regions, did not find differences in factors affecting job retention in ‘difficult’ regions between those in Dakar with experience in ‘difficult’ regions and those currently working in ‘difficult’ regions [14].

## Conclusion

The study used a CE to elicit the job-related preferences of physician and non-physician health workers in ‘difficult’ regions of Senegal. For both groups, provision of a permanent contract, the availability of equipment in health facilities and the provision of training opportunities are the most valued rural work conditions. This is the first study to look at the impact of different contract types on the retention of health workers in rural areas. Contract type—either permanent or non-permanent—was found to be a key factor in rural job retention. Indeed, this result has led the Senegalese Health Ministry to introduce a rural assignment policy that recruits permanent staff from the pool of annually contracted healthcare professionals on the condition that the workers are assigned to rural posts. While our results suggest that this is a useful policy development, they also suggest that further policy development is required to ensure sufficient numbers of health workers in underserved areas to guarantee equitable access to quality healthcare for the people in those communities. A combination of individual incentives and health system improvements would facilitate the retention of health professionals in rural jobs.

## Abbreviations

BWS: Best-worst scaling
CE: Choice experiment
GP: General practitioners
LMICs: Low- and middle-income countries
MNL: Multinomial logit (MNL)
SE: Standard errors
UHC: Universal Health Coverage
WTS: Willingness to stay
